# Identification of severe acute respiratory syndrome coronavirus 2 breakthrough infections by anti-nucleocapsid antibody among fully vaccinated non-healthcare workers during the transition from the delta to omicron wave

**DOI:** 10.3389/fmed.2022.1019490

**Published:** 2022-11-29

**Authors:** Yu-Ching Dai, Yen-Chia Lin, Lauren L. Ching, Alanna C. Tseng, Yujia Qin, Vivek R. Nerurkar, Wei-Kung Wang

**Affiliations:** ^1^Department of Tropical Medicine, Medical Microbiology and Pharmacology, John A. Burns School of Medicine, University of Hawai‘i at Mānoa, Honolulu, HI, United States; ^2^Pacific Center for Emerging Infectious Diseases, John A. Burns School of Medicine, University of Hawai‘i at Mānoa, Honolulu, HI, United States; ^3^Department of Quantitative Health Sciences, John A. Burns School of Medicine, University of Hawai‘i at Mānoa, Honolulu, HI, United States

**Keywords:** severe acute respiratory syndrome coronavirus 2, variants of concern, breakthrough infection, neutralizing antibodies, anti-nucleocapsid antibody, whole genome sequencing

## Abstract

Uncontrolled transmission of severe acute respiratory syndrome coronavirus 2 (SARS-CoV-2) has led to the emergence of several variants of concern (VOC). As vaccine-induced neutralizing antibodies against VOC waned over time, breakthrough infections (BTIs) have been reported primarily among healthcare workers or in long-term care facilities. Most BTIs were identified by reverse transcription-polymerase chain reaction (RT-PCR) or antigen test for individuals experiencing symptoms, known as symptomatic BTIs. In this study, we detected seroconversion of anti-nucleocapsid (N) antibody to identify both symptomatic and asymptomatic BTIs in a cohort of COVID-19-naive university employees and students following two or three doses of mRNA vaccines. We reported 4 BTIs among 85 (4.7%) participants caused by the Omicron and Delta VOC during the transition from the Delta to Omicron wave of the pandemic; three were symptomatic and confirmed by RT-PCR test and one asymptomatic. A symptomatic reinfection two and half months after a BTI was found in one participant. Two of three symptomatic BTIs and the reinfection were confirmed by whole genome sequencing. All were supported by a >4-fold increase in neutralizing antibodies against the Delta or Omicron variant. Moreover, we found both symptomatic and asymptomatic BTIs can boost neutralizing antibodies against VOC with variable degrees ranging from 2.5- to 77.4-fold increase in neutralizing antibody titers. As BTIs continue, our findings highlight the application of anti-N antibody test to ongoing studies of immunity induced by spike-based vaccine, and provide new insights into the establishment of herd immunity in the community during the post-vaccination era.

## Introduction

Unconstrained transmission of severe acute respiratory syndrome coronavirus 2 (SARS-CoV-2) has resulted in the emergence of several variants of concern (VOC), including previously circulating VOC, the Alpha (Pango lineage: B.1.1.7), Beta (B.1.351), Gamma (P1), and Delta (B.1.617.2) variants, and the currently circulating VOC, the Omicron (B.1.1.529) variant and Omicron subvariants under monitoring such as BA.4, BA.5, and BA.2.75 (1, 2). Genomic surveillance of variants revealed that the Delta variant rose from <1% of circulating viruses in the US in early May 2021 to >50% in June, and to >95% in all 10 regions by August 2021. Since the first US Omicron case reported on 12/1/2021, the Omicron variant increased from >1% of circulating lineages in early December, to >50% in late December, and to >99% in late January 2022 (3, 4). The proportion of circulating variants in Hawaii, belonging to region 9, followed a similar trend.

In agreement with reports that vaccine-induced neutralizing antibodies against SARS-CoV-2 reduced and waned over time, breakthrough infections (BTIs) have been well documented following two or three doses of mRNA vaccines (5–16). Studies of BTI have been primarily focused on healthcare workers or residents in long-term care facilities (5, 14, 17–20). Based on reverse transcription-polymerase chain reaction (RT-PCR) results, Rana et al. reported a BTI rate of 2.0% among 3650 healthcare workers following the second dose of vaccine (14). Similarly, Bergwerk et al. reported a BTI rate of 2.7% among 1497 fully vaccinated healthcare workers (5). The BTI rates in long-term care homes were reported to be 39.6 and 9.3% among fully vaccinated residents and staff, respectively, during an outbreak of Gamma variant, and 6.5 to 50% among fully vaccinated residents during the Beta variant outbreak (17, 19, 20). The extent and rate of BTIs among non-healthcare workers remain understudied. The most common method to identify BTIs is RT-PCR or antigen test for individuals experiencing symptoms, thereby identifying symptomatic BTIs. Detection of anti-nucleocapsid (N) protein antibody has potential to identify both symptomatic and asymptomatic BTIs among COVID-19-naive vaccinees who received a spike (S)-based vaccine such as mRNA vaccines (21). Although recent studies reported potent and broad neutralizing antibodies induced by BTIs (22–26), how a BTI boosts neutralizing antibodies against VOC in individuals remains incompletely understood. In this study, we employed a combination of anti-N and anti-S enzyme-linked immunosorbent assays (ELISAs) to investigate BTI in a cohort of university employees and students following two or three doses of mRNA vaccines and examine neutralizing antibodies before and after BTI in Hawaii.

## Materials and methods

### Human subjects

With the approval of Institutional Review Board of the University of Hawaii (2020-00406), coded plasma samples were obtained with informed consents from study participants (university employees and students), who were COVID-19-naïve or recovered cases at enrollment and received two or three doses of mRNA-1273 (Moderna) or BNT162b2 (Pfizer) vaccine between August 2020 and February 2022 (27, 28). The samples included COVID-19-naïve participants before vaccination (*n* = 28), COVID-19-naïve participants following one (*n* = 55), two (*n* = 147), or three (*n* = 20) doses of a mRNA vaccine, RT-PCR-confirmed cases of SARS-CoV-2 natural infection (NI) before vaccination (*n* = 19), and NI followed by one (*n* = 10) or two (*n* = 7) doses of a mRNA vaccine (Table 1). All participants were otherwise healthy adults based on a self-reported questionnaire.

**TABLE 1:** Characteristics of study participants.

Characteristics^a^	Naïve vaccinees pre-vaccine	Naïve vaccinees post-dose 1	Naïve vaccinees post-dose 2	Naïve vaccinees post-dose 3	NI vaccinees pre-vaccine	NI vaccinees post-dose 1	NI vaccinees post-dose 2
No: subjects/samples	28/28	53/55	85/147	20/20	19/19	10/10	7/7
Sampling days^b^ (days, mean [range])	NA^c^	20.3 [13–41]	96.2 [12–278]	29.8 [13–91]	127 [13–340]	15.1 [6–27]	25.3 [14–58]
Vaccine type (mRNA-1273 [Moderna]/ BNT162b [Pfizer])	NA^c^	36/17	47/37	10/10	NA^c^	1/9	1/6
Age (years, mean [range])	38.4 [20–76]	39.4 [20–85]	39.9 [20–85]	46.7 [20–85]	49.6 [21–78]	53.7 [25–72]	49.9 [27–66]
Gender (male/female)	14/14	23/30	36/49	8/12	8/11	4/6	4/3
Race/ethnicity (A/W/mixed/Others/Hispanic)^d^	14/12/1/1/1	23/23/5/2/2	45/31/6/3/5	13/7/0/0/0	7/7/3/2/1	3/4/3/0/1	2/3/2/0/0

^a^COVID-19-naïve; NI, natural infection.

^b^Sampling days after RT-PCR positive for NI and after dose 1, 2, or 3 for vaccinee panels.

^c^NA, not applicable.

^d^A: Asian, W: White, Others: Native Hawaiian, non-native Hawaiian Pacific Islanders and Black.

### SARS-CoV-2 ELISAs

Anti-N and anti-S antibodies were detected by SARS-CoV-2 Detect IgG ELISA (InBios) and Platelia SARS-CoV total Ab ELISA (BioRad), respectively.

### Plasmids

Plasmids pNL4-3 R-E-miRFP, which contains the miRFP gene replacing the Luc gene of an *env*-defective HIV-1 reporter construct pNL4-3.Luc.R- E-, and D614G, which contains the S gene of the SARS-CoV-2 Wuhan-Hu-1 strain with D614G mutation and C-terminal 19-residue truncation, have been described previously (28). The S genes (Alpha, Delta) were synthesized (Integrated DNA Technologies) by two fragments [residues 1 to 461, flanked by *Kpn*I and *Afl*II (an introduced silent site mutation) sites, and residues 461 to 1254, flanked by *Afl*II and *Not*I sites], and cloned into the plasmid D614G (with *Kpn*I and *Not*I sites) by 3-fragment ligation to generate plasmid Alpha. Two-step cloning (residues 1 to 461 first, followed by residues 461 to 1254) and one-step cloning of four fragments (residues 1 to 461, residues 461 to 853, and residues 853 to 1254) by NEBuilder HiFi DNA assembly kit (New England Biolab) were performed to generate plasmids Delta and Omicron, respectively. All plasmids were confirmed by sequencing of the entire S gene insert (Supplementary Figure 1) and verified for expression by transfection and Western blot analysis (28, 29).

### Generation of SARS-CoV-2 pseudovirus

To generate pseudoviruses, HEK-293T cells were seeded in 10-cm dish 1 day before transfection, co-transfected with pNL4-3 R-E-miRFP (12 μg) and S plasmid (3 μg) using lipofectamine 2000, and incubated with DMEM media containing 10% FBS (28). The supernatants were collected at 48 h post transfection, followed by low-speed centrifugation at 300 × *g* for 10 min, aliquoted and stored at −80°C. Previously, 1.65 × 10^9^ RNA copies of pseudovirus per well were used for each neutralization test, resulting in miRFP signals 10 times higher than mock-infected well at 72 h post-infection (28). To titrate each pseudovirus, threefold serially diluted supernatants were inoculated to HEK-293ThACE2 cells by spin infection (28); miRFP signals were quantitated at 72 h post-infection, and the amount of pseudovirus that resulted in miRFP signals 10 times higher than the mock-infected wells was used for neutralization test. Pseudovirus D614G was referred as the wild type D614G strain, which predominated since March 2020 and contains the S gene similar to that of the USA-WA1 strain used in the mRNA vaccine with one amino acid substitution (G at residue 614) (2).

### SARS-CoV-2 pseudovirus neutralization test

HEK-293T-hACE cells (2 × 10^4^ cells/well) were seeded onto 96-well plates 1 day before infection. Pseudovirus (D614G, Alpha, Delta, or Omicron variant) was mixed with fourfold serial dilutions of plasma at 1:1 ratio, incubated at 37°C for 1 h, and added to each well for spin infection. At 72 h, the plates were scanned by Li-Cor Odyssey imager (28). The % of infection at different plasma dilutions (from 1:10 to 1:10,240 dilutions) were calculated by the formula (intensity of plasma + pseudovirus—intensity of media only)/(intensity of pseudovirus only—intensity of media only) × 100. The % neutralization = 100 −% of infection (28). NT_50_ titer was the plasma dilution that reached 50% neutralization using 4-parameter non-linear regression analysis (GraphPad 6.0) (Supplementary Figure 2). NT_50_ titer <10 was arbitrarily assigned as 5.

### Processing of nasal swabs

Nasal swabs were collected between 2 and 5 days following symptom onset and stored in virus transport medium (VTM). SARS-CoV-2 infection was confirmed by quantitative RT-PCR using the CDC 2019-Novel Coronavirus (2019-nCoV) Real-Time RT-PCR Diagnostic Panel.

### Whole genome sequencing

Total RNA was extracted from 200 μl of VTM using the MagMax™ Viral/Pathogen II kit with the Kingfisher™ Flex Purification System. RNA was reverse transcribed into cDNA using random hexamers (SuperScript™ IV first-strand synthesis system, Life Technology). PCR amplification of the SARS-CoV-2 genome was adapted from the ARTIC network^1^ sequencing protocol (30) using the ARTIC V4.1 primers. Purified PCR amplicons were submitted to the Advanced Studies in Genomics, Proteomics and Bioinformatics (ASGPB) core at the University of Hawai‘i at Mānoa for library preparation and sequencing. Sequencing libraries were prepared using the Nextera XT kit according to the manufacturer’s instructions and sequenced using the Illumina MiSeq V3 platform.

### Whole genome sequencing and analysis

The consensus sequences from the reads were generated by the nf-core/viralrecon pipeline (31), using the options for the Illumina amplicon-based library protocol. Consensus genome sequences were submitted to GenBank after validation using VADR SARS-CoV-2 models (32). The genomes were classified into lineages using Pangolin (33) and into clades using Nextclade (34).

### Statistical analysis

The two-tailed Mann–Whitney test was used to compare quantitative variables between two groups (GraphPad 6.0).

## Results

**FIGURE 1:**
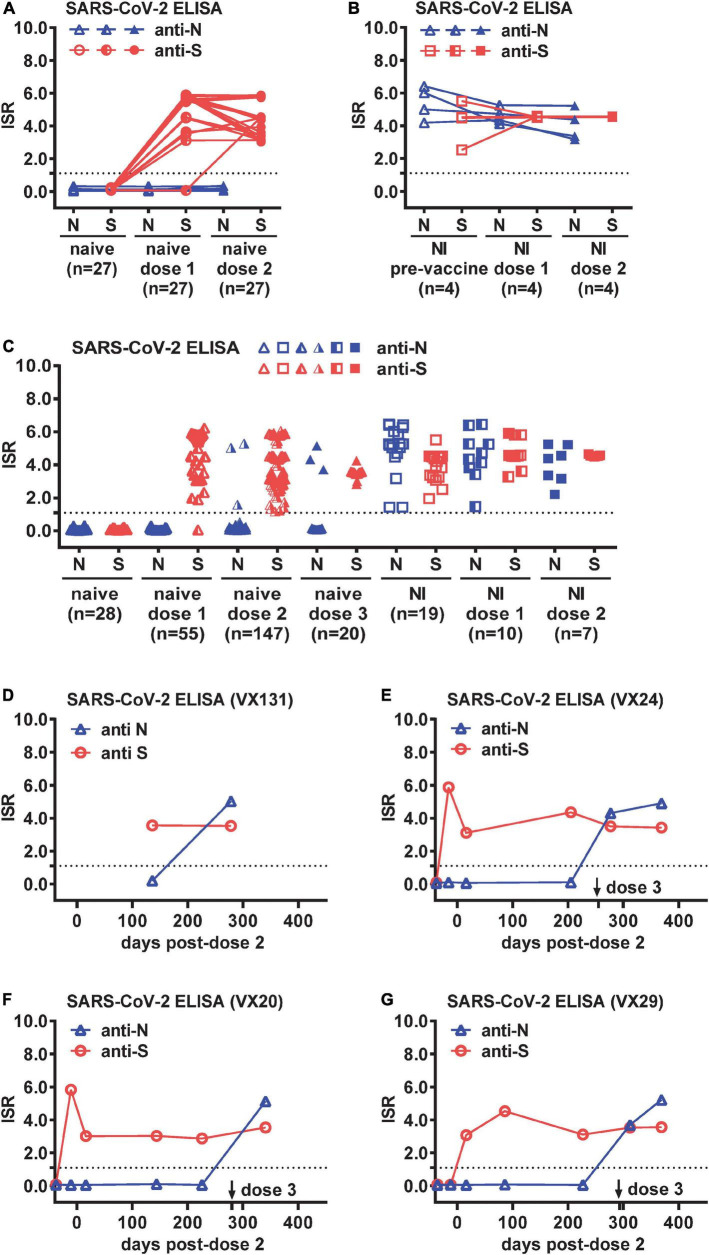
Breakthrough infections (BTIs) identified by anti-N and anti-S ELISAs among COVID-19-naïve vaccinees. Results of anti-N and anti-S ELISAs of sequential plasma samples from **(A)** COVID-19-naïve (*n* = 27) and **(B)** SARS-CoV-2 natural infection (NI) (*n* = 4) panels before and after one and two doses of a mRNA vaccine (Moderna or Pfizer). **(C)** Results of anti-N and anti-S ELISAs of single or sequential plasma samples from COVID-19-naïve participants following one (*n* = 55), two (*n* = 147), or three (*n* = 20) doses of a mRNA vaccine, and controls from COVID-19-naïve (*n* = 28), NI (*n* = 19), and NI followed by one (*n* = 10) or two (*n* = 7) doses of a mRNA vaccine. **(D–G)** Results of anti-N and anti-S ELISAs of sequential plasma samples from four COVID-19-naïve participants (VX131, VX24, VX20, VX29) with a BTI identified by anti-N antibody seroconversion following two or three doses of Moderna vaccine. Dotted lines indicate the cut-off of immunological status ratio (ISR) values for ELISAs. Data are the mean of duplicates from one experiment.

To examine the feasibility of using anti-N and anti-S ELISAs to distinguish COVID-19-naïve, COVID-19-naïve vaccinees, and COVID-19-recovered cases with or without vaccination in our study which involved S-based vaccines only, we first tested sequential samples from COVID-19-naive participants (*n* = 27) and participants with RT-PCR-confirmed SARS-CoV-2 NI (*n* = 4), including pre-vaccination and ∼2 weeks following one or two doses of mRNA (Moderna or Pfizer) vaccine. As expected, neither anti-S nor anti-N antibody was detectable in the naïve group before vaccination, and anti-S but not anti-N antibody was detected following administration of one (26/27 positive) or two (27/27 positive) doses of a mRNA vaccine (Figure 1A). Both anti-S and anti-N antibodies were detected in the NI group and within the NI group after one or two doses of mRNA vaccines (Figure 1B).

We next tested larger panels to identify BTIs, including samples from COVID-19-naïve participants following one (*n* = 55), two (*n* = 147), or three (*n* = 20) doses of a mRNA vaccine, as well as controls from COVID-19-naïve (*n* = 28), NI (*n* = 19), and NI followed by one (*n* = 10) or two (*n* = 7) doses of a mRNA vaccine (Table 1). The age, gender and sampling days following vaccination in each panel were comparable except that the NI post-dose 1 panel were older compared with the naïve post-dose 1 or 2 panel (*p* = 0.02, two-tailed Mann–Whitney test) and had shorter sampling days after vaccination compared with the naïve post-dose 1, 2, or 3 panel (*p* = 0.02, 0.0007, or 0.002, respectively, two-tailed Mann–Whitney test) (Table 1). Of the 222 samples from 85 COVID-19-naïve participants, anti-N antibody was detected in six participants following two or three doses of a mRNA vaccine (Figure 1C). While two of them had no earlier samples available, suggesting a possible BTI, the other four had earlier samples (anti-N antibody negative) to demonstrate anti-N antibody seroconversion, indicating a BTI (Figures 1D-G). Of the four BTIs, one was after the second dose and three after the third dose. The four BTIs occurred between November 2021 and February 2022, which was during the transition from the Delta to Omicron wave of the pandemic.

**FIGURE 2:**
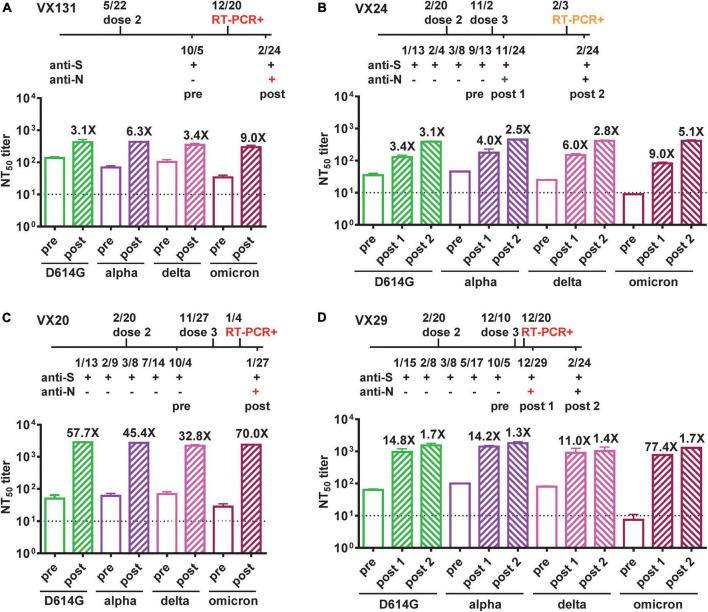
Neutralizing antibodies against VOC before and after BTIs. **(A–D)** Time course of vaccination, anti-N and anti-S ELISAs, and RT-PCR test of four COVID-19-naïve participants (VX131, VX24, VX20, VX29) who received two or three doses of Moderna vaccine with a BTI identified by anti-N antibody seroconversion. Symptomatic BTIs with positive RT-PCR test and anti-N ELISA are shown in red, asymptomatic BTI with positive anti-N ELISA in blue, and symptomatic reinfection with positive RT-PCR test in orange. NT_50_ titers against D614G, Alpha, Delta and Omicron variants before (pre, open bars) and after (post, hatched bars) BTIs were determined by pseudovirus neutralization test (28). Number x = fold increase in NT_50_ titers compared with previous time point. Dotted lines indicate NT_50_ titer = 10. Data are the means and standard deviations of duplicates from one experiment.

We further performed pseudovirus neutralization test to assess neutralizing antibodies against the wild type D614G strain and the Alpha, Delta and Omicron VOC before and after BTI for the four participants, and compared with time course including symptoms and RT-PCR results. Participant VX131 received the second dose of Moderna vaccine on 5/5/2021, started to have COVID-19 symptoms >7 months later with an RT-PCR positive test on 12/20/2022; the symptomatic BTI was supported by anti-N antibody seroconversion 2 months later (2/24/2022) and the NT_50_ titers showed 3.1 to 9.0-fold increase with a >4-fold increase against the Omicron variant (9.0-fold), the predominant VOC at the time (Figure 2A). Participant VX24 received the third dose of Moderna vaccine on 11/2/2021 and had anti-N antibody seroconversion on 11/24/2021 without apparent symptoms, suggesting an asymptomatic BTI, which was supported by a >4-fold increase in NT_50_ titers against the Delta variant (6.0-fold), the predominant VOC then (Figure 2B). Two and half months later, VX24 developed COVID-19 symptoms with an RT-PCR positive test (2/3/2022), suggesting a new and symptomatic reinfection; the NT_50_ titers 3 weeks later (2/24/2022) revealed a >4-fold increase against the predominant Omicron variant (5.1-fold) then (Figure 2B). Participant VX20, who received the third dose of Moderna vaccine on 11/27/2021, started having symptoms 1 month later with an RT-PCR positive test (1/4/2022); the symptomatic BTI was supported by anti-N antibody seroconversion 3 weeks later (1/27/2022) and a >4-fold increase in NT_50_ titers against the predominant Omicron variant at the time. Interestingly, the NT_50_ titers showed a 32.8 to 70.0-fold increase against the four variants tested (Figure 2C), suggesting stronger booster effect of BTI on neutralization titers compared with the first two cases. Participant VX29, who received the third dose of Moderna vaccine on 12/10/2021, started having symptoms 1 week later with an RT-PCR positive test (12/20/2022); the symptomatic BTI was supported by anti-N antibody seroconversion 9 days later (12/29/2022) and a >4-fold increase in NT_50_ titers against the predominant Omicron variant. Notably, the NT_50_ titers revealed a 11.0 to 77.4-fold increase against the four variants tested (Figure 2D). Follow-up NT_50_ titers 2 months later (2/24/2022) revealed a slightly further increase (1.3 to 1.7-fold) against the four variants.

To confirm the variants responsible for BTIs and reinfection, we conducted whole genome sequencing of 2 available RT-PCR positive samples from the 4 BTI cases and one from the reinfection case. The identified variants were BA.1.1.2 (VX-131) and BA.1.1 (VX-29) for BTIs and BA.1.1 (VX-24) for reinfection (Table 2).

**TABLE 2:** Severe acute respiratory syndrome coronavirus 2 (SARS-CoV-2) variants confirmed by whole genome sequencing.

Study participants^a^	Type of infection^b^	Collection date	Clade^c^	Pango lineage^c^	GenBank accession
VX131	symptomatic BTI	12/20/2021	21K (Omicron)	BA.1.1.2	ON103223
VX29	symptomatic BTI	12/20/2021	21K (Omicron)	BA.1.1	ON103174
VX24	reinfection	2/3/2022	21K (Omicron)	BA.1.1	ON103202
VX20	symptomatic BTI	N/A	N/A	N/A	N/A
VX24	asymptomatic BTI	N/A	N/A	N/A	N/A

^a^For time course of the study participants refers to Figure 2.

^b^BTI: breakthrough infection, N/A: not available.

^c^Whole genome sequencing of SARS-CoV-2 variants was conducted from available RT-PCR positive nasal swab samples of BTIs and reinfection.

## Discussion

In this study, we report that two simple IgG ELISAs can distinguish COVID-19-naïve (both anti-N and anti-S negative), COVID-19-naïve vaccinees (anti-S positive only), or COVID-19-recovered cases with or without vaccination (both anti-N and anti-S positive) in places where only S-based vaccines, such as mRNA, adenovirus-vectored, and recombinant S protein vaccines are implemented. With available sequential samples, anti-N antibody seroconversion can identify BTI among COVID-19-naïve vaccinees (anti-S positive and anti-N seroconversion).

Using anti-N antibody to identify BTIs, Laing et al. reported a BTI rate of 0.88% for symptomatic infection and 25% for asymptomatic infection in 227 healthcare workers (21). Others reported BTI rates of 0.6 and 0.8% among 4111 and 130 fully vaccinated healthcare workers, respectively (35, 36). Another study reported a BTI rate of 4.6 and 5.3% in patients with immune-mediated inflammatory diseases with (*n* = 3207) and without immunosuppressants (*n* = 985), respectively, and 4.0% in 822 healthy controls (37). Our finding of a BTI rate of 4.7% among 85 fully vaccinated non-healthcare workers was similar to the reported rate of healthy controls.

Interestingly, we found that BTIs have variable booster effects on neutralizing antibodies against VOC among the four participants, all young female (21 to 27 years old), healthy without immunocompromised conditions and receiving the Moderna vaccine. Of the four participants, one (VX131) had a BTI after the second dose, three had BTIs after the third dose, either at 1 month (VX20) or <3 weeks (VX24 and VX29) following the third dose. Due to the short time interval between the third dose and BTI, there was no sampling during this window to distinguish between the booster effects of the third dose of mRNA vaccine and BTI. Nonetheless, variable booster effects of BTI plus the third dose were observed; VX24 had a 3.4 to 9.0-fold increase in NT_50_ titers against the four variants tested, whereas VX20 and VX29 had a 32.8 to 70.0-fold and 11.0 to 77.4-fold increase in NT_50_ titers, respectively.

Since the variants responsible for BTIs in this study were only confirmed in two out of four by genome sequencing, they were all assessed by the predominant circulating variant and supported by neutralization test, which showed a >4-fold increase in NT_50_ titers against the predominant variant at the time (Figure 2). It is worth noting that despite previous studies reported the booster effect of BTI on NT_50_ titers was not specific to the circulating variant, a >4-fold increase in NT_50_ titers against the variant causing BTI was always observed (23, 38). Consistent with the reported intervals between infection and re-infection, 46 days to 6 months (39), VX24 experienced a symptomatic reinfection during the Omicron wave two and half months after the BTI, which was supported by a 5.1-fold increase in NT_50_ titers against the Omicron variant (Figure 2B). A recent study reported a Delta variant BTI followed by reinfection with the Omicron variant in a fully vaccinated healthcare worker (40); our study reports a similar case of BTI followed by reinfection in the community, which may reflect what was happening in the real world. Notably, the confirmation of the BA.1.1 and BA.1.1.2 subvariants in two BTIs on 12/20/2021 by whole genome sequencing was in agreement with reports of these subvariants during the initial phase of Omicron wave (2).

With the widespread transmission of the Omicron VOC and its subvariants since late 2021, it is likely that BTIs and reinfections occur commonly as supported by the rapid rise of the seroprevalence of anti-N antibody (41, 42). Given the vaccine-induced and infection-induced immunity together with the attenuated replication and pathogenicity of the Omicron VOC, most BTI and reinfections resulted in asymptomatic infection or mild disease, however, the concern of new VOC with increased virulence remains (43–46). Although an increase in anti-N antibody seroprevalence could compromise the detection of BTIs by anti-N antibody alone, future studies using anti-N antibody test (IgG and/or IgM) in combination with RT-PCR or antigen test could still identify BTIs among COVID-19-naïve vaccinees (anti-N negative during the window period before seroconversion) and distinguish them with reinfection among COVID-19-recovered cases (anti-N positive). Investigation of the extent of BTIs and reinfections in the community would provide new insights into future vaccine and booster strategies to combat the morbidity and mortality caused by next VOC and further our understanding of how herd immunity is built in the post-vaccination era. As BTIs, which has been shown to boost immunity (22–26), continue, an immediate application of our study is to use anti-N antibody test and/or RT-PCR or antigen test to identify BTIs in ongoing COVID-19 vaccine studies aiming to explicit the breadth and durability of vaccine-induced immunity.

There were several limitations. First, the sample size was small. Nonetheless, comparing with other studies using anti-N antibody to identify BTI within the same period as this study, the BTI rate (4.7%) based on 85 participants in our study was similar to the BTI rate (4.0%) based on 822 healthy controls in one study and lower than that (25%) based on 227 healthcare workers in another study (21, 37). Second, anti-N antibody test cannot be used to identify SARS-CoV-2 infection (either NI, BTI or reinfection) in countries where inactivated COVID-19 vaccines such as Covaxin, which contains the N protein, are implemented. Nonetheless, recent studies have shown anti-ORF8 and ORF3b antibodies as new serological markers of SARS-CoV-2 infection in these countries (47, 48). Third, since these BTIs occurred when Omicron, and subvariants BA.1, BA1.1, and BA.2 were circulating, which have been shown to be antigenically equidistant from the wild-type virus and shared similar neutralization profiles, neutralization test was performed against the parental Omicron VOC (B.1.1.529) only but not the BA.4 or BA.5 subvariant (49, 50). Fourth, as the peak of IgG antibody was reported to be at 2 and 3 weeks following the second dose of mRNA vaccine and infection, respectively (12, 51), we measured the NT_50_ titers within 3 weeks following BTIs, presumably the peak of IgG, in three cases (VX24, VX20, and VX29), and 2 months following BTI in another case (VX131).

## Data availability statement

The original contributions presented in this study are included in the article/Supplementary material, further inquiries can be directed to the corresponding author.

## Ethics statement

The studies involving human participants were reviewed and approved by the Institutional Review Board of the University of Hawai’i (2020-00406). The patients/participants provided their written informed consent to participate in this study.

## Author contributions

WKW, VRN, YCD, YCL, LLC, ACT, and YQ contributed to study design, experiments, and data analysis. WKW, VRN, and LLC contributed to sample collection and manuscript writing. All authors contributed to the article and approved the submitted version.

## Footnotes

1
https://artic.network/ncov-2019
