# Supplementary material for: Identification of severe acute respiratory syndrome coronavirus 2 breakthrough infections by anti-nucleocapsid antibody among fully vaccinated non-healthcare workers during the transition from the delta to omicron wave
Source: Front Med (Lausanne). 2022 Nov 29;9:1019490. doi: 10.3389/fmed.2022.1019490 (PMC9745036; doi:10.3389/fmed.2022.1019490)
Supplement: Supplementary file 1 [file Data_Sheet_1.PDF]

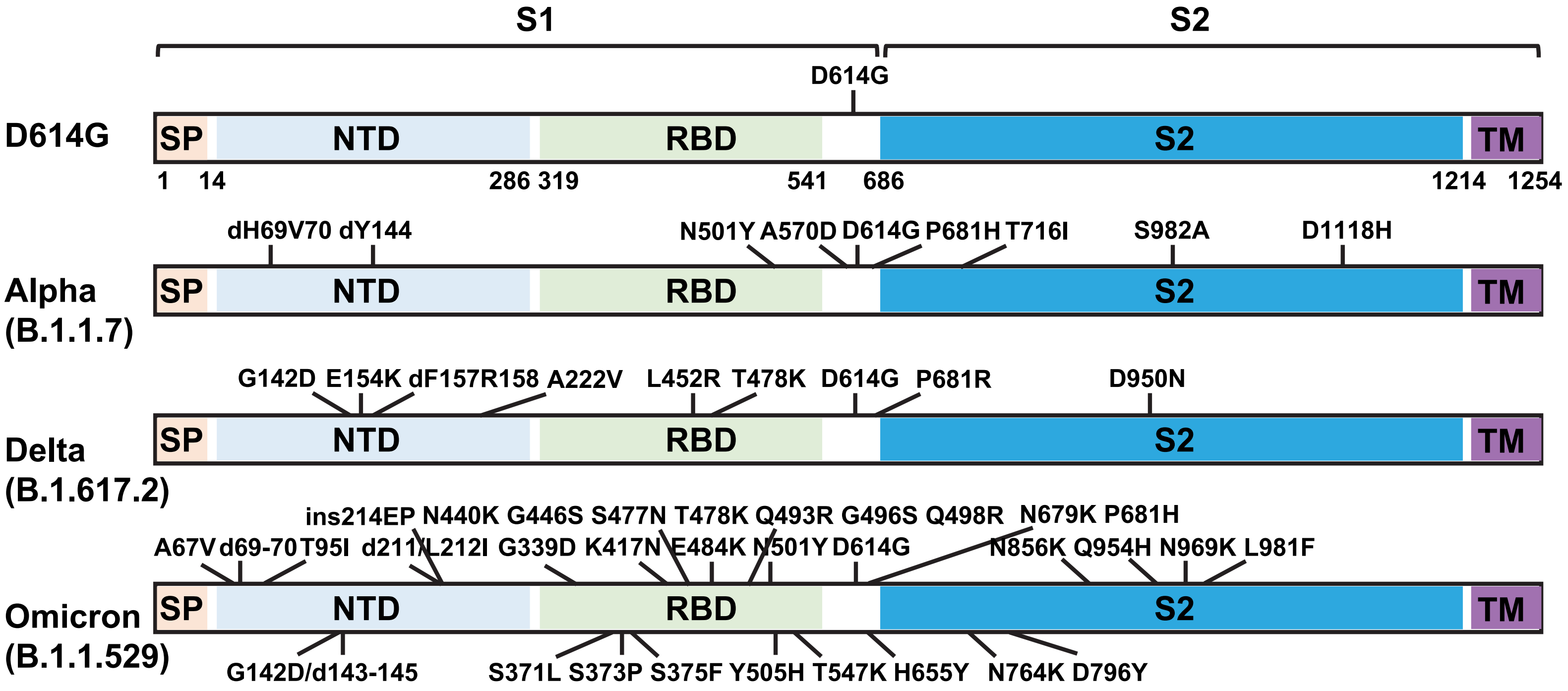

FIGURE S1 | Plasmids expressing the S proteins of the D614G, alpha, delta and omicron VOC and their mutations generated in this study. SP, signal peptide; S1 and S2, S1 and S2 subunits of the S protein; NTD, N-terminal domain; RBD, receptor binding domain; TM, transmembrane domain (29).

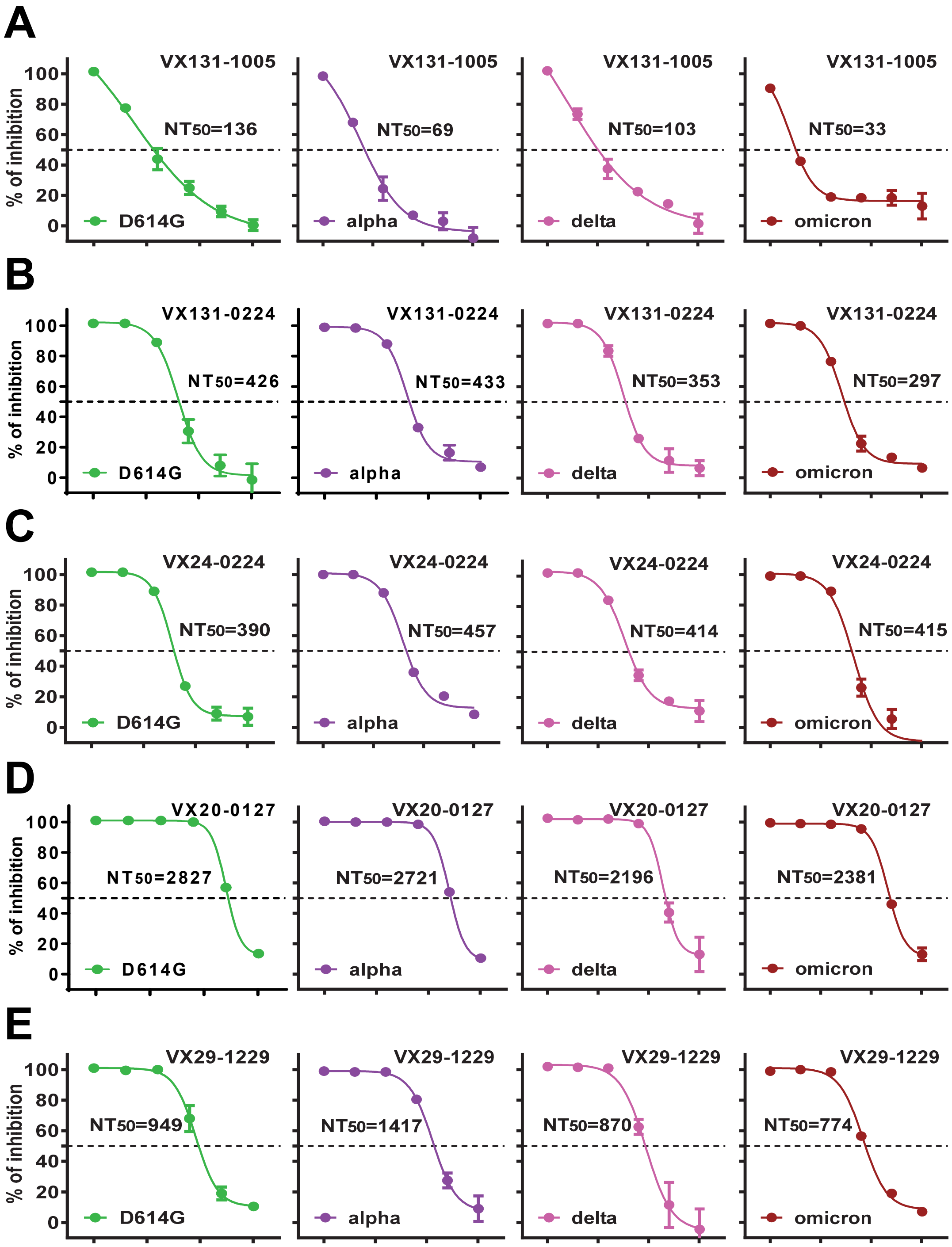

FIGURE S2 | Neutralization curves and NT_{50} titers against VOC in four COVID-19-naïve participants with a BTI following two or three doses of Moderna vaccine. (**A,B**) VX131 before (**A**) and after (**B**) BTI. (**C**) VX24 after BTI and reinfection. (**D,E**) VX20 (**D**) and VX29 (**E**) after BTI. Data are the means standard deviations of duplicates from one experiment.
